# Proteomic analysis of *Taenia solium* cysticercus and adult stages

**DOI:** 10.3389/fvets.2022.934197

**Published:** 2023-01-09

**Authors:** Lizhu Li, Wei He, Xianmin Fan, Meichen Liu, Bo Luo, Fengjiao Yang, Nan Jiang, Lingjun Wang, Biying Zhou

**Affiliations:** Department of Parasitology, School of Basic Medical Sciences, Zunyi Medical University, Zunyi, China

**Keywords:** cysticercus, host-parasite interaction, mass spectrometry proteomic analysis, *Taenia solium*, parallel reaction monitoring

## Abstract

*Taenia solium* (*T. solium*) cysticercosis is a neglected parasitic zoonosis that occurs in developing countries. Since *T. solium* has a complex life cycle that includes eggs, oncospheres, cysticerci, and adults, presumably many proteins are produced that enable them to survive and establish an infection within the host. The objectives of this study were to perform a comparative proteomic analysis of two ontogenetic stages of *T. solium* (cysticerci and adult) and to analyze their differential expression of proteins. Methods proteins were separated by High Performance Liquid Chromatography (HPLC) fractionation, and protein samples were also digested in liquid and identified by liquid chromatography tandem mass spectrometry (LC-MS/MS); the differentially expressed proteins were then processed by a bioinformatics analysis and verified by parallel reaction monitoring (PRM). Results we identified 2,481 proteins by label-free quantitative proteomics. Then differentially expressed proteins were screened under *P* values < 0.05 and 2 fold change, we found that 293 proteins up-regulated and 265 proteins down-regulated. Discussion through the bioinformatics analysis, we analyzed the differences types and functions of proteins in the *Taenia solium* and cysticercus, the data will provide reference value for studying the pathogenic mechanism of the two stages and the interaction with the host, and also support for further experimental verification.

## Introduction

Cysticercosis is caused by the larvae (cysticercus) of *Taenia solium* and is one of the 20 neglected tropical diseases according to the World Health Organization (WHO) (1). The life cycle of *T. solium* is complex, however, requiring the parasitism of two kinds of mammals, namely, pigs and humans, which serve as intermediate hosts, whereas only the latter are the final hosts. The transmission of *T. solium* from a tapeworm carrier occurs *via* the shedding of eggs in feces, followed by their ingestion by hosts (e.g., pigs and humans) and subsequent development into cysticercus (2).

In the past 10 years, vaccines and drugs have been jointly administered to pigs followed by strategic deworming treatments of infected humans (3). Because of the complex life cycle of tapeworms, they have developed strategies to protect themselves at each specific stage, thus providing favorable conditions for their survival. For example, the activity of its metabolic enzymes is related to growth and development. In addition, cestodes have evolved specific detoxification pathways to absorb nutrients from the host as a source of their metabolic energy (4). The adult stage causes *T. solium* taeniasis in humans, and the larval stage (cysticerci) causes porcine and human cysticercosis. It can cause neurocysticercosis and seizures (5). *T. solium* cysts are known to induce persistent infections that can last for decades in humans. Establishing such long-term infections requires modulating the host immune system for a long period of time and suggests the existence of a vast array of immunoregulatory mechanisms (6, 7). This is associated with the exposure and intensity of infection as well as the developmental stage of the parasite and induced immune response. It may also be related to the inhibitory effect of T regulatory cells (8–10). Until now, however, it is unknown which parasitic molecules are responsible for these effects.

Mass spectrometry-based proteomics can detect and compare thousands of proteins on a large scale, contributing to the understanding of the functional role of proteins in biological systems. Proteomics of worms had been shown to understand their biology/pathogenesis, diagnostic biomarkers, new drug targets, or potential vaccine candidates. Several reports that investigated the proteome of *T. solium* across its developmental stages have discovered that cytoskeleton, actin, and paramyosin can be used as targets for applied research on cysticercosis vaccines and potential diagnostic antigens (e.g., enolase, calcium-binding protein, small molecule shock protein, 14-3-3 protein, tropomyosin α, α-1 tubulin, β tubulin, annexin B1, and cAMP cyclic adenylate protein-dependent kinase) (11–14). However, we expect to increase our knowledge of their pathogenesis through the *T. solium* and cysticercus proteomes.

Accordingly, in this study, we sought to compare the proteomes of two consecutive developmental stages, finding both similarities and differences between *T. solium* adults and larvae. These differences in protein activity may be crucial for influencing the parasite's invasion, survival, immune evasion, and worm development. The major goal of our study is to provide data to support experiments to understand the pathogenic mechanisms of *T. solium* adults and larvae.

## Materials and methods

### Parasites

A whole adult *T. solium* was obtained from a patient with taeniasis from a taeniasis-endemic area in Yajiang, Ganzi, Sichuan Province. To generate *T. solium* larvae, each healthy piglet was infected by feeding 5 pieces of mature gravid proglottids from the worm at the Animal Experimental Center of Zunyi Medical University. There were 12 piglets in this infection experiment, including 10 piglets in the infection group and 2 piglets in the control group. The healthy piglets were confirmed to be pathogen-free before infection and raised under standard conditions (15). The infected piglets were euthanized 2–3 months postinfection, and *Cysticercus cellulosae* were harvested from the muscle tissue. After obtaining the parasite, we observed its structure under a microscope.

### Protein extraction

The *T. solium* was washed 3 times with phosphate-buffered saline (PBS), cut into pieces using scissors, ground and divided into 1.5 ml centrifuge tubes, and centrifuged at 4°C and 12,000 r/min for 5 min, and the supernatant was collected. The *C. cellulosae* were collected and washed three times with physiological saline and PBS, and then homogenized in a homogenizer (70 Hz/s, 10 s/time) at 4°C until completely lysed. The supernatants were collected, and their protein concentration was measured using a Bradford protein assay kit (Beyotime, China) according to the manufacturer's instructions and stored at −80°C until further use.

Each protein sample was first ground with liquid nitrogen into cell powder and then transferred into a 5-ml centrifuge tube. Next, four volumes of lysis buffer (8 M urea; Sigma, China) and 1% protease inhibitor (Calbiochem, Germany) were added to the cell powder, followed by sonication three times on ice using a high-intensity ultrasonic processor (Scientz); the remaining debris was removed by centrifugation at 12,000 *g* for 10 min at 4°C. Finally, the supernatant was collected and the protein concentration was determined using a BCA kit (Beyotime, China) according to the manufacturer's instructions.

### Trypsin digestion

The protein solution was added with dithiothreitol to a final concentration of 5 mM and incubated for 30 min at 56°C. Then, iodoacetamide was added to a final concentration of 11 mM and incubated for 15 min at room temperature in the dark. Finally, the urea concentration of the sample was diluted to less than 2 M. Trypsin was added at a mass ratio of 1:50 (trypsin: protein) and digested overnight at 37°C. The trypsin was added at a mass ratio of 1:100 and was digested for 4 h and continued for 4 h.

### HPLC fractionation

The tryptic peptides were separated into fractions by high pH reverse-phase HPLC, using a Thermo Betasil C18 column (5-μm particles, 10-mm inner diameter, 250-mm length). Briefly, peptides were first separated by a gradient spanning 8 to 32% acetonitrile (pH 9.0) over 60 min, which generated sixty fractions. Then, these peptides were pooled into six fractions and dried by vacuum centrifuging.

### LC-MS/MS analysis

The tryptic peptides were dissolved in 0.1% formic acid (solvent A) and directly loaded onto a homemade reversed-phase analytical column (15-cm length, 75-μm inner diameter.). The gradient consisted of an increase from 6 to 23% solvent B (0.1% formic acid in 98% acetonitrile) over 26 min, followed by 23 to 35% over 8 min and climbing to 80% in 3 min, then holding it at 80% for the last 3 min, all performed at a constant flow rate of 400 nl/min on an EASY-nLC 1000 UPLC system. The peptides were subjected to a nanoelectrospray ionization (NSI) source, followed by tandem mass spectrometry (MS/MS) in a Q ExactiveTM Plus (Thermo) coupled online to the UPLC system. The electrospray voltage applied was 2.0 kV; the m/z scan range was 350 to 1,800 for each full scan, and intact peptides were detected in the Orbitrap at a resolution of 70,000. Peptides were then selected for MS/MS by using an NCE set to 28, with fragments detected in the Orbitrap at a resolution of 17,500. A data-dependent procedure was used, one that alternated between a single MS scan followed by 20 MS/MS scans with a 15.0-s dynamic exclusion. The automatic gain control (AGC) was set to 5E4. The fixed first mass was set to 100 m/z.

### Database search

The secondary mass spectrum data were searched using Maxquant (version 1.5.2.8, http://www.maxquant.org/). For the retrieve parameter settings, the database is *Taenia*_*asiatica*_60517_PR_20190708 (10,328 sequences). Trypsin/P was specified as the cleavage enzyme, permitting up to four missing cleavages. The mass tolerance for precursor ions was set to 20 ppm in the “First search” and 5 ppm in the “Main search,” while the mass tolerance for fragment ions was set to 0.02 Da. Carbamidomethyl on Cys was specified as the fixed modification and acetylation modification and oxidation on Met were specified as the variable modifications. The false discovery rate (FDR) was adjusted to < 1% and a minimum score for modified peptides was set *a priori* to >40.

### Bioinformatics

#### GO annotation

Gene ontology (GO) annotation (http://www.maxquant.org/) of the parasite's proteome was based on the UniProt-GOA database (http://www.ebi.ac.uk/GOA/). First, each identified protein ID was converted into a UniProt ID and then mapped to the existing GO IDs by protein ID. For those identified proteins that could not be annotated by the UniProt-GOA database, the InterProScan software was used to annotate the GO function of the proteins based on the protein sequence alignment method. Then, all the proteins were, respectively, classified by GO annotation according to their three main categories, namely, biological process (BP), cellular component, and molecular function.

#### Enrichment of pathways

The Encyclopedia of Genes and Genomes (KEGG, http://www.genome.jp/kaas-bin/kaas_main) database was used to identify enriched pathways by applying a two-tailed Fisher's exact test to test the enrichment of the differentially expressed protein against all the identified proteins. The pathway with a corrected *P-*value < 0.05 was considered significant. These pathways were classified into hierarchical categories, as recommended on the KEGG website.

#### Mass spectrometry-based targeted proteomic quantification by parallel reaction monitoring

The tryptic peptides were dissolved in 0.1% formic acid (solvent A) and directly loaded onto a homemade reversed-phase analytical column. The gradient was comprised of an increase from 6 to 23% solvent B (0.1% formic acid in 98% acetonitrile) over 38 min, 23 to 35% in 14 min, and climbing to 80% in 4 min and then holding at 80% for the last 4 min, all at a constant flow rate of 700 nl/min on an EASY-nLC 1,000 UPLC system. The peptides were subjected to an NSI source followed by tandem mass spectrometry (MS/MS) in Q ExactiveTM Plus (Thermo) coupled online to the UPLC system.

The electrospray voltage applied was 2.0 kV. The m/z scan range was 350 to 1,000 for the full scan, and intact peptides were detected in the Orbitrap at a resolution of 35,000. Peptides were then selected for MS/MS using the NCE setting as 27 and the fragments were detected in the Orbitrap at a resolution of 17,500. A data-independent procedure alternated between one MS scan followed by 20 MS/MS scans. AGC was set at 3E6 for full MS and 1E5 for MS/MS. The maximum IT was set at 20 ms for full MS and auto for MS/MS. The isolation window for MS/MS was set at 2.0 m/z.

## Results

### Protein identification analysis of differentially expressed proteins by LC-MS/MS

**Figure 1:**
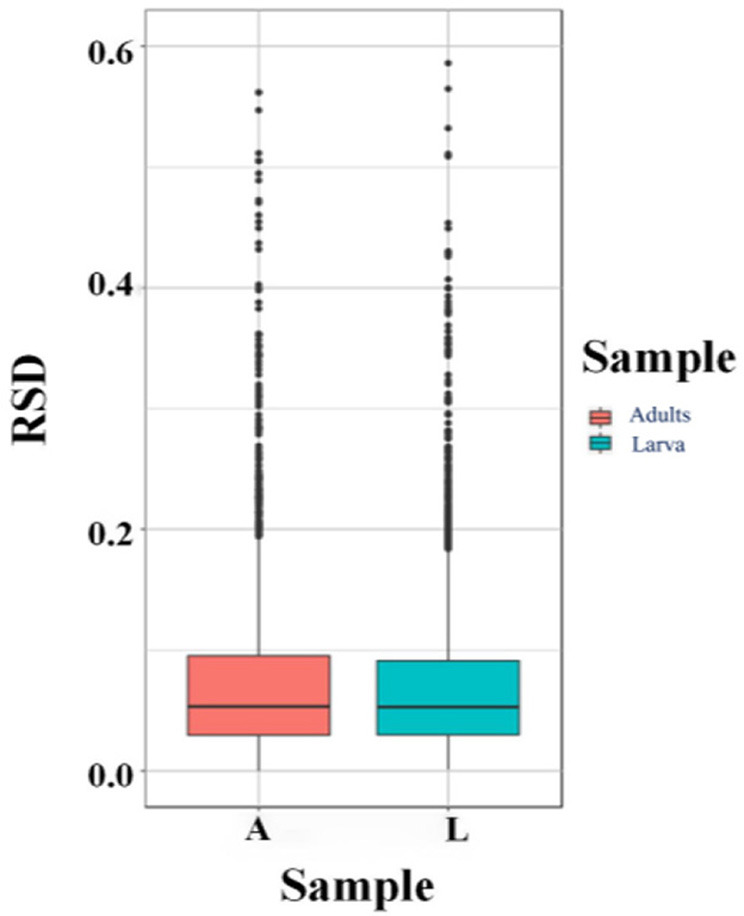
Relative standard deviations (RSDs). A, adults; L, Larva (cysticercus).

According to MS/MS analysis, the total peptide identified was 143,698, the specific peptide was 19,210, and a total of 3,658 proteins were identified, of which 2,481 contained quantitative information (Tables 1, 2). Relative standard deviation (RSD) was used to estimate repeatability, and a lower RSD (*n* = 3) value increases the accuracy of the repeatability (Figure 1). A corrected *P*-value of 0.05 and minimum fold-change of 2 were set as threshold criteria for determining the significant differential expression of a protein. In comparing the *T. solium* adults and larvae, we found 293 upregulated proteins (adults) and 265 downregulated proteins (cysticerci) (Supplementary Table S1).

**Table 1:** MS/MS spectrum database search analysis summary.

**Total spectrum**	**Matched spectrum**	**Peptides**	**Unique peptides**	**Identified proteins**	**Quantifiable proteins**
942,271	223,234 (23.7%)	30,689	29,761	3,658	2,481

**Table 2:** Differentially expressed protein summary (filtered according to a threshold value of expression fold-change with a *P*-value < 0.05); A = adult, L = larva.

**Compared groups**	**Regulated type**	**Fold-change > 2**
A/L	Upregulated	293
	Downregulated	265

### Protein functional classification based on gene ontology of differentially expressed proteins in *Taenia solium* and cysticercus stage

The identified proteins were classified according to their molecular function, cellular component, and BP. Among these differentially expressed proteins, many were found to be involved in cellular processes, biological regulation, metabolic processes, multicellular organismal processes, developmental processes, and immune system processes. The predominant molecular functions are related to binding (e.g., protein binding) and catalytic activity (hydrolase and oxidoreductase activity). Regarding BPs, the predominant number of proteins is involved in metabolic and cellular processes. For cellular components, our GO results indicated that the identified proteins are primarily associated with cell parts, intracellular, and protein-containing complexes.

The forty downregulated (cysticercus stage) proteins were most enriched in terms of regulation of immune system process at biological function; such as protein kinase C, E3 ubiquitin-protein ligase, SH2 domain-containing protein, annexin, cAMP-dependent protein kinase regulatory, and fatty acid binding protein (Supplementary Table S2). Both annexin and human plasminogen proteins are known for their strong antigenicity, hence, their use as diagnostic antigens. In addition, 25 other proteins involved as immune effectors were found (Supplementary Table S3).

**Figure 2:**
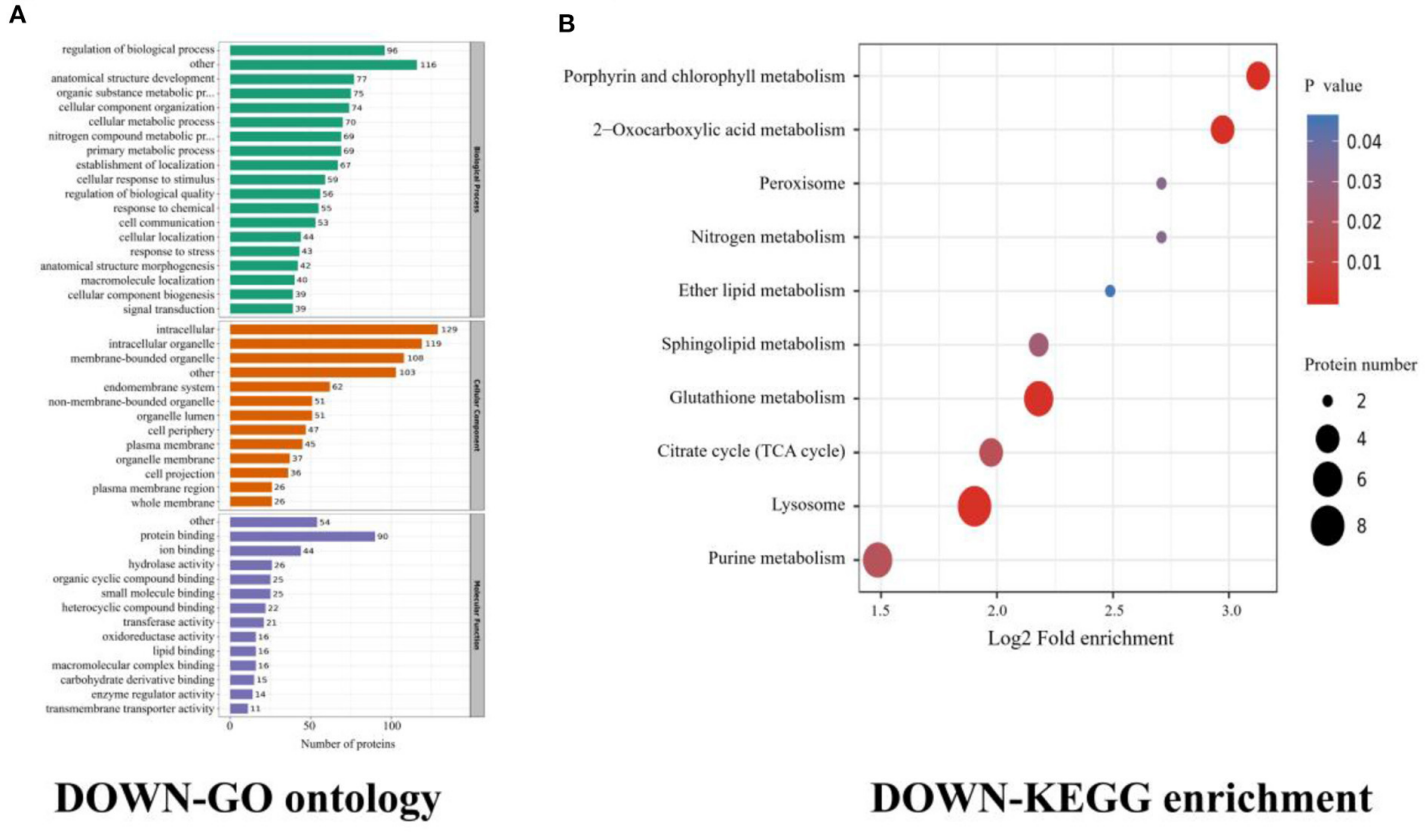
Cysticercus (downregulated) proteins GO ontology and KEGG enrichment. **(A)** Gene ontology (GO) of cysticercus, dividing the annotated proteins into 3 categories: cellular component, molecular function, and biological process. The number of genes is expressed as the number of annotation proteins. **(B)** KEGG enrichment analysis of identified proteins in cysticercus.

The results revealed that most downregulated proteins were enriched in the regulation of protein localization to membrane, bicarbonate transport, neuroepithelial cell differentiation, and mechanoreceptor differentiation, all under the BP category. The molecular function was enriched for cytoskeletal protein binding and actin binding. The cellular component was enriched in terms of the intracellular vesicle, cytoplasmic vesicle, plasma membrane region, and secretory vesicle (Figure 2A).

**Figure 3:**
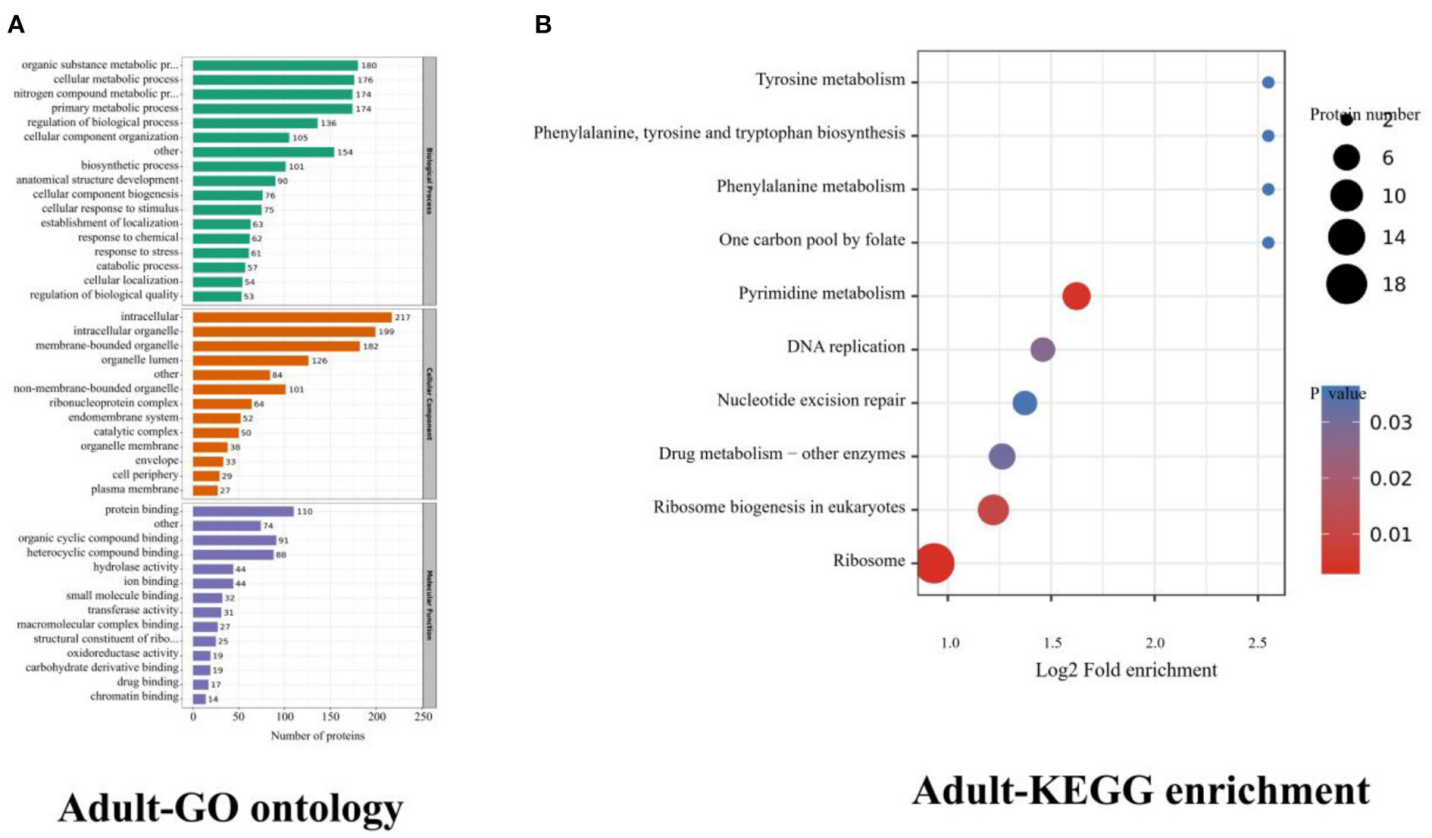
Adults (upregulated) proteins GO ontology and KEGG enrichment. **(A)** Gene ontology (GO) of adults, dividing the annotated proteins into 3 categories: cellular component, molecular function, and biological process. The number of genes is expressed as the number of annotation proteins. **(B)** KEGG enrichment analysis of identified proteins in adults.

The upregulated (adult stage) proteins were mainly enriched in terms of regulation of metabolic processes in biological function (Figure 3A). Most proteins are involved in the metabolic process, of which nineteen of these proteins have been studied in other parasites, all of which are related to the growth, development, and metabolism of parasites, and only 2 proteins are related to immune function (Supplementary Tables S4, S5).

### KEGG enrichment of differentially expressed proteins in *Taenia solium* and *cysticercus* stage

In the cysticercus stage, the KEGG enrichments included lysosome, glutathione metabolism, porphyrin and chlorophyll metabolisms, and 2-oxocarboxylic acid metabolism (Figure 2B, Table 3). Isocitrate dehydrogenase [NADP], NAD_binding_2 domain-containing protein, 6PGD domain-containing protein, and phospholipid hydroperoxide glutathione were involved in glutathione metabolism; 5-aminolevulinate synthase, porphobilinogen deaminase, coproporphyrinogen III oxidase, and protoporphyrinogen oxidase were involved in porphyrin and chlorophyll metabolisms; isocitrate dehydrogenase [NADP], aconitate hydratase, and citrate synthase were involved in 2-oxocarboxylic acid metabolism; ADP ribosylation factor-binding protein GGA1, beta-galactosidase, Prosaposin a preproprotein, and LITAF domain-containing protein were involved in the lysosome.

**Table 3:** KEGG enrichment of differentially expressed proteins in cysticercus.

**KEGG pathway**	**Protein accession**	**Protein description**	**Ratio**	***p* value**
Lysosome	A0A0R3WB48	ADP ribosylation factor binding protein GGA1 (predicted)	0.356	0.0001596
	A0A0R3W2H9	Peptidase A1 domain-containing protein (predicted)	0.343	3.825E-05
	A0A0R3W9I1	Beta-galactosidase	0.332	3.743E-05
	A0A0R3W6D3	SSD domain-containing protein (predicted)	0.495	0.0001026
	A0A0R3W4P0	Prosaposin a preproprotein (predicted)	0.265	1.792E-06
	A0A0R3VUB5	LITAF domain-containing protein (predicted)	0.496	0.0004954
	A0A0R3WF80	ML domain-containing protein (predicted)	0.152	4.469E-05
	A0A0R3VWZ7	Cathepsin L-like cysteine peptidase (predicted)	0.25	0.0004831
Glutathione metabolism	A0A0R3W597	Sigma-type glutathione S-transferase (predicted)	0.133	2.071E-05
	A0A0R3WGD8	6PGD domain-containing protein (predicted)	0.336	1.574E-05
	A0A0R3WDK3	Phospholipid hydroperoxide glutathione (predicted)	0.376	0.0002813
	A0A0R3W2C6	Glutathione transferase (predicted)	0.233	4.331E-06
	A0A0R3WC84	Isocitrate dehydrogenase [NADP]	0.018	2.789E-07
	A0A0R3VXS3	NAD_binding_2 domain-containing protein (predicted)	0.4	0.00028
Porphyrin and chlorophyll metabolism	A0A0R3WES1	5-aminolevulinate synthase	0.259	0.010716
	A0A0R3W2K4	Porphobilinogen deaminase (predicted)	0.249	4.424E-05
	A0A0R3W1Y3	Coproporphyrinogen III oxidase (predicted)	0.112	8.166E-05
	A0A0R3W877	Protoporphyrinogen oxidase	0.36	2.51E-06
2-Oxocarboxylic acid metabolism	A0A0R3VW15	Aconitate hydratase, mitochondrial	0.27	3.231E-06
	A0A0R3W1A1	Citrate synthase	0.086	1.44E-09
	A0A0R3WA42	Aminotran_1_2 domain-containing protein (predicted)	0.17	0.0002166
	A0A0R3WC84	Isocitrate dehydrogenase [NADP]	0.018	2.789E-07

In the adult stage, the KEGG enrichments included ribosome, pyrimidine metabolism, and ribosome biogenesis in eukaryotes. There were 18 proteins involved in the ribosome, CTP synthase, PNP_UDP_1 domain-containing protein, and Thymidylat_synt domain-containing protein were associated with pyrimidine metabolism; ribonucleoside-diphosphate reductase subunit and ribonucleoside-diphosphate reductase were involved in glutathione metabolism and pyrimidine metabolism. 39S ribosomal protein L3, 60S ribosomal protein L3, Ribosomal_L2_C domain-containing protein, 40S ribosomal protein S4, Ribosomal protein L11, Ribosomal S15a protein, Ribosomal protein l5, large subunit ribosomal protein l7e, and 39S ribosomal protein L12 were associated with the ribosome biogenesis in eukaryotes (Figure 3B, Table 4).

**Table 4:** KEGG enrichment of differentially expressed proteins in the adult stage.

**KEGG pathway**	**Protein accession**	**Protein description**	**Ratio**	***p* value**
Ribosome	A0A0R3WCV3	39S ribosomal protein L12 (predicted)	2.028	0.0027817
	A0A0R3VVI0	Ribosomal protein L11 (predicted)	2.117	0.0002648
	A0A0R3W3C5	40S ribosomal protein S4	2.346	1.86E-05
	A0A0R3W4M0	40S ribosomal protein S15 (predicted)	2.16	0.0006813
	A0A0R3VSK8	60S ribosomal protein L3 (predicted)	2.105	1.671E-05
	A0A0R3W1U3	60S ribosomal protein L6 (Fragment) (predicted)	3.029	0.0001604
	A0A0R3VVJ5	Ribosomal S15a protein (predicted)	2.287	4.257E-06
	A0A0R3W373	Large subunit ribosomal protein l7e (predicted)	2.233	0.0010246
	A3F4S0	60S ribosomal protein L18	2.934	8.693E-07
	A0A0R3WDH4	60S ribosomal protein L35a (predicted)	2.078	0.0002394
	A0A0R3W3L7	60S ribosomal protein L36 (predicted)	2.887	0.0007586
	A0A0R3VT58	Ribosomal protein L15	2.288	0.000801
	A0A0R3WC08	Ribosomal_L2_C domain-containing protein (predicted)	2.27	0.0019432
Pyrimidine metabolism	A0A0R3W9Z3	PNP_UDP_1 domain-containing protein (predicted)	6.624	4.536E-09
	A0A0R3W9X3	Ribonucleoside diphosphate reductase subunit (predicted)	25.427	4.19E-06
	A0A0R3W5N1	Purine nucleoside phosphorylase	544.455	0.0002366
	A0A0R3WBK6	Thymidylat_synt domain-containing protein (predicted)	5.608	0.033756
	A0A0R3WB77	Purine nucleoside phosphorylase	11.818	0.0036187
	A0A0R3W2V2	Ribonucleoside-diphosphate reductase	30.579	0.0001017
	A0A0R3WDN1	CTP synthase	2.942	4.126E-05
Ribosome biogenesis in eukaryotes	A0A0R3WAR2	Ribosome biolocus tagsis protein BMS1 (predicted)	5.45	0.036556
	A0A0R3WCI1	rRNA 2 O methyltransferase fibrillarin (predicted)	2.055	4.133E-05
	A0A158R6D7	RNA cytidine acetyltransferase	2.041	0.0182639
	A0A0R3WDJ3	Nuclear valosin containing protein (predicted)	2.264	0.0002803
	A0A0R3W7X6	AAA family ATPase (predicted)	2.651	0.000219
	A0A0R3W830	Ribosomal_L7Ae domain-containing protein (predicted)	2.032	0.0001434
	A0A0R3VW23	OBG-type G domain-containing protein (predicted)	2.274	5.622E-05
	A0A0R3WB31	Nop domain-containing protein (predicted)	2.214	7.687E-05

### Differentially expressed protein quantification by mass spectrometry-based targeted proteomics

**Figure 4:**
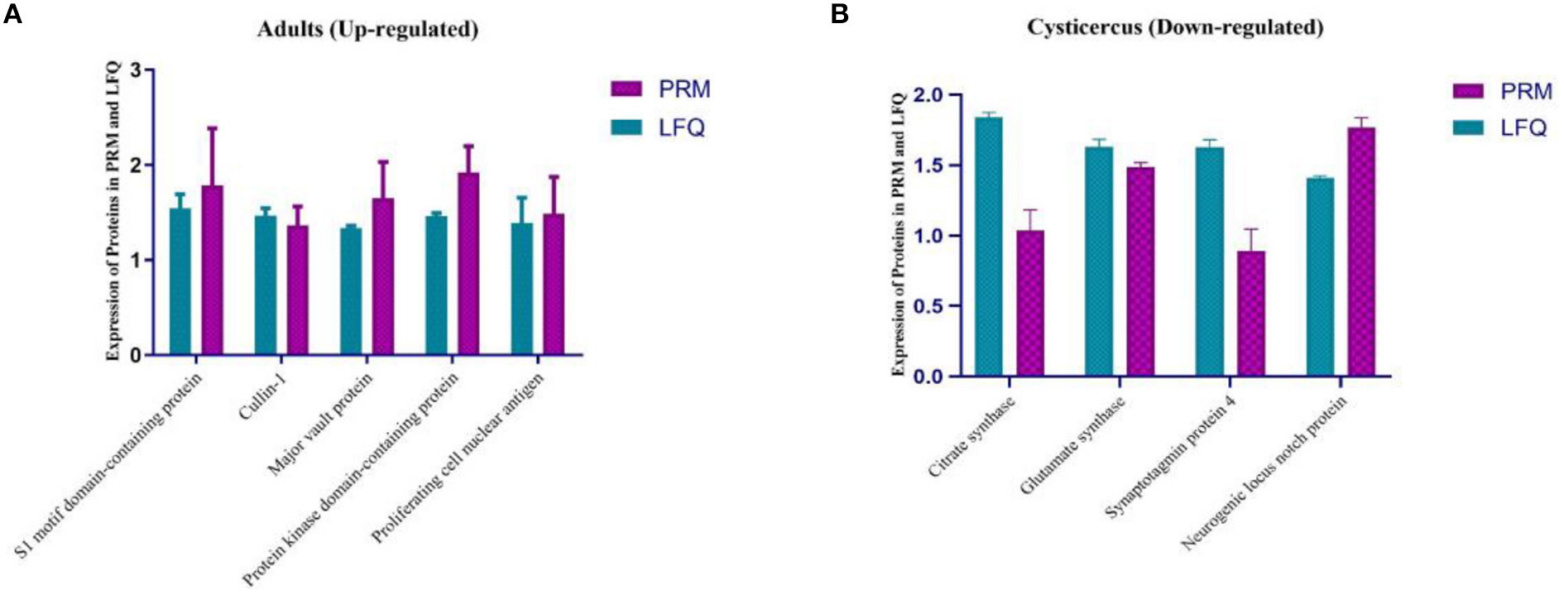
**(A, B)** Differentially expressed protein quantification by mass spectrometry-based targeted proteomics (PRM). PRM, parallel reaction monitoring; LFQ, lab-free quantitation.

According to GO annotation and KEGG enrichments, we analyzed 9 out of 20 differentially expressed proteins with PRM instead of traditional western blotting. The quantitative information of PRM protein was calculated according to the ion peak area of the peptide. Four proteins were identified in adults (Figure 4A), namely, S1 motif domain-containing protein, Cullin-1, Protein kinase domain-containing protein, and proliferating cell nuclear antigen. Four proteins were identified in the larvae (Figure 4B), namely, citrate synthase, glutamate synthase, synaptotagmin protein 4, and neurogenic locus notch protein.

## Discussion

Examination of homologous genes among human *Taenia* tapeworms showed that 11,888 (90.3%) *T. asiatica* genes had homologs in *Taenia saginata* and *T. solium*. However, *T solium* lacks a complete proteomic database, and we performed sequence alignments between the proteins of *T. solium*'s cysticercus and adult stages by comparing them to the *T. asiatica* database—corresponding to specific proteins of this species and searching the library according to the sequence of more than one specific peptide of the protein and analyzed them using bioinformatics. Although the survival of either the cysticercus or adult depends on the energy metabolism in its host, their main energy source, carbohydrates, might be catabolized by aerobic respiration or by two complementary anaerobic pathways, those of lactate fermentation and malate dismutation (4). In addition, both rely on glutathione metabolism to grow and develop in the host. The difference is that adults can also survive through pyrimidine metabolism. Glutathione plays a role in antioxidant defense and in maintaining the reducing environment of the cytosol; many known glutathione-dependent processes are directly related to the specific lifestyle of the parasite. In the *Plasmodium falciparum*, proteins involved in GSH-dependent processes are studied as factors in the pathophysiology of malaria but also as potential drug targets (16). In KEGG enrichment of cysticercus and adults, pyrimidine and purine are the raw materials for the synthesis of DNA and RNA. The parasite itself synthesizes pyrimidine and forms its own set of survival mechanisms, therefore, generating new lead compounds to treat malaria and schistosomes by targeting purine and pyrimidine pathways (17, 18). Glutathione-related enzymes were identified in the proteomics of both adults and larvae, while proteins involved in pyrimidine metabolism were only identified in adults. Pyrimidine metabolic pathway can be used as an idea for the treatment of taeniasis, which also shows the differences in growth and development between adults and larvae.

Analyzing the genomes of four species of tapeworms, the researchers found that there were a large number of genes lost in the genome in order to adapt to parasitic life, but some genes that can increase the survival rate of the parasite were amplified simultaneously. For example, the tapeworm genome lacks genes related to synthesizing fatty acids and cholesterol *de novo* (19, 20). Over time, tapeworms have lost their ability to synthesize essential fats and cholesterol essential for larval development, which they obtain from their hosts. However, there are a large number of lipid elongating enzymes and fatty acid transporter genes, and a large number of fatty acid-binding proteins and apolipoprotein B antigens are expressed, which may be related to the parasite consuming a lot of nutrients in the process of growth and development. These proteins have been identified in the proteomics of both adults and larvae.

The transcriptome is used to study the expression and regulation level of functional gene mRNA, which is highly sensitive to the detection of differentially expressed genes and can more truly reflect the whole process of complex expression regulation of transcriptome. Through the transcriptome analysis of *T. solium* cysticerci, it was found that paramyosin, major egg antigen, cathepsin L-like cysteine proteinase, heat shock protein 70 kDa protein, and H17g or TEG-Tsol surface antigen (21) have the potential to develop worm antigens for immunodiagnosis or vaccine (22). Similar to the results of proteomic analysis, most of the proteins are also involved in cell process, catalytic activity, and binding proteins. The selection of diagnostic antigens was based on the cloning of genes encoding the antigens or the screening of recombinant antigens from the *T. solium* cDNA library, which is relevant to the identification of different regions, developmental stages, and body parts of *T. solium* (21, 23–27). Transcriptome can observe the expression and regulation level of mRNA at different time points and observe the related pathways more intuitively. Proteomics is the identification of the whole protein in the sample and can only observe static proteins in sample preparation. On the basis of the transcriptome, it can have a more comprehensive understanding of the pathogenic mechanism and treatment of C. cellulosae.

### Identified proteins in the cysticercus (downregulated)

Through a functional enrichment analysis, we found 25 proteins involving the immune process in the larva. It included known proteins such as thioredoxin peroxidase, calcium-binding protein 39, fatty acid-binding protein FABP2, and protein kinase C. Calcium-binding protein is involved in various regulatory functions of host invasion by parasites, and these mainly include members of the calmodulin family (CAM), the calcineurin B-like (CBL) family, and the calcium-dependent protein kinase (CDPK) family: all of these are highly conserved helix–loop–helix structure, namely, the EF-hand domain. In *Plasmodium* and *Toxoplasma*, phosphatidylinositol can also be stimulated by ligands binding to surface receptors (such as G-protein binding receptors [GPCR]), to produce calcium signals, thereby stimulating multiple cellular pathways (28). Fatty acid-binding proteins (FABPs) are a family of proteins with binding of triclabendazole (29), anti-oxidant activity, and immunomodulation (30). It is used as a potential drug target in *Echinococcus granulosus* and schistosomiasis (31, 32). Some proteins have been studied for immunity in other parasitic worms. For example, the *Trichinella spiralis* ES product thioredoxin peroxidase-2 induces macrophages toward an M2-like phenotype, both *in vivo* and *in vitro*, and CD4^+^T cells increased in number after immunization of mice with rTsTPX2 and mediate the expulsion of the worm from the host to protect them, thus suggesting TsTPX2 is a potential vaccine candidate against trichinosis (33). Also, thioredoxin peroxidase was significantly recognized by melioidosis-positive sera in the cysticercus stage, for which strong immunogenic properties render it an anticipated vaccine target (34). Pyruvate kinase is a crucial glycolytic enzyme that has been characterized in *Clonorchis sinensis* (35) and it promotes the development of Th1 and inhibition of dendritic cells (DCs).

Some researchers revealed that fructose-1-6-bisphosphate aldolase (FBPA) proteins of *T. solium* cyst fluid were mainly enriched PI3K-Akt signaling pathways which are thought to play important roles in proliferation, migration, invasion, and drug resistance (36, 37). FBPA is a crucial glycolytic enzyme and a plasminogen-binding protein and is involved in parasitic motion and invasion by connecting surface adhesion proteins to the actin-myosin of parasites (38). Specifically, FBPA binds to the surface of the cell membrane, and then binds to plasminogen, using the activity of hydrolyzing surface-related proteins to help invade host cells; hence, it is also considered a potential vaccine candidate or chemotherapy target for *Trichinella spiralis* and *Giardia* infections (39–41).

Furthermore, antigens and immunomodulatory proteins observed in cysticercus are able to play a special mechanism of adaptation at this developmental stage, one that has evolved to evade the host immune system, which is a prerequisite for establishing a successful infection. We have only discussed the known proteins, and there are still several unknown proteins with specific functions that need to be verified.

### Identified proteins in the adults (upregulated)

Lacking a digestive tract, *T. solium* instead absorbs nutrients from its host across the body wall. The cortex absorbs all kinds of nutrients by means of diffusion and active transport, and it also has the function of secreting and resisting the destruction of the host's digestive juice. Their main energy source, carbohydrates, can be catabolized by aerobic respiration or by two complementary anaerobic pathways, namely, lactate fermentation and malate dismutation (4). In this study, we found the upregulated proteins, most of the proteins are related to metabolism. Following PRM analysis, we found that the expression of four proteins (S1 motif domain-containing protein, Cullin-1, protein kinase domain-containing protein, and proliferating cell nuclear antigen) was consistently elevated, and several proteins whose functions are lacking in the study of the porcine tapeworm have important values in other parasite fields, which provides us with different insights into the tapeworm. Cullin protein is the most typical ubiquitin ligase family and a key tumor-associated protein, which is able to promote the proliferation of tumor cells and also can be used as a marker and therapeutic target for tumor prognosis. According to the GO analysis, the Cullin protein is also involved in cell cycle progression, signal transduction and transcription, intracellular metabolism, and the corresponding response to stimuli. However, this protein has not been studied in parasites and could be a research target to be verified in the next experiments (42–44). Proliferating cell nuclear antigen (PCNA) is a protein that acts as a processivity factor for DNA polymerase δ in eukaryotic cells and was originally identified as an antigen expressed in the nuclei of cells during the DNA synthesis phase of the cell cycle (45, 46). The major vault protein is a ribonucleoprotein that is highly conserved in lower and higher eukaryotes, and its function is not clear (47). Some studies have found that *Schistosoma mansoni* participates in the adaptation mechanism of parasitic hosts in the process of infection.

Topoisomerase is a critical type of enzyme for overcoming the problem of chromosome topology that arises during DNA replication, transcription, recombination, and mitosis, and they are involved in cell growth, tissue development, and cell differentiation (48–50). These enzymes participate in a variety of DNA metabolic processes; in the parasitic infection, the inhibition of topoisomerase II by different ketone benzene and furan derivatives in cells hinders the basic metabolic process of cells and eventually leads to apoptosis. In view of the parasite DNA topoisomerase II, these compounds can be used as potential antiparasitic drugs. *Schistosome* parasite miRNA mediates the activity of the frizzled protein (frizzled-related protein 1), which increases liver fibrosis, in that FRZB2 is a secreted frizzled protein-related protein, which can competitively bind to specific frizzled protein receptors to suppress the signal transduction of Wnt, thus affecting the severity of liver fibrosis. The FRZB2 gene knockdown affected *S. japonicum* morphology, development, survival, as well as reproductive capacity (51, 52). *Plasmodium* glyceraldehyde-3-phosphate dehydrogenase functions as a glycolytic enzyme and also as a host plasminogen-binding protein, thereby interacting with the host's fibrinolytic system to establish an important mechanism for a parasite's invasion, growth, and development. At the same time, it can be used as a potential diagnostic biomarker for a variety of parasites, such as *Plasmodium, T. solium, E. granulosus*, filariasis, *S. mansoni*, and *Babesia microti* (53–57). The distinct mechanisms of cell cycle arrest are associated with the upregulating or downregulating of TbPCNA. Deregulating the intra-parasite levels of TbPCNA is a potential strategy for therapeutically exploiting this target in the bloodstream form of *Trypanosoma brucei*, which shows that PCNA is related to that parasite's growth and development (58).

Adults only survive in the host's intestines and escape the immune response without causing excessive damage to the host. In our study, we found that there are few proteins related to immunity, and most of them are related to growth and development, which also reflects the difference between adults and larvae to the host.

## Conclusion

Cysticercosis/taeniosis remains a serious neglected public health problem in many developing countries. Even if there are preventive and therapeutic approaches, we need to be constantly updated it. Proteomics now gives us a broader platform for research. In the previous several years, tremendously useful advances were made in the field of proteomics in terms of rapid and sensitive technologies, which provided greater proteome coverage. We profiled the proteome of *T. solium*'s cysticercus and adult stages. We analyzed the different types and functions of proteins in *T. solium* and cysticercus. The data will provide a reference value for studying the pathogenic mechanism of the two stages and the interaction with the host, as well as support for further experimental verification.

## Data availability statement

The datasets presented in this study can be found in online repositories. The names of the repository/repositories and accession number(s) can be found in the article/Supplementary material.

## Ethics statement

The animal study was reviewed and approved by Zunyi Medical University's ethical review of experimental animals.

## Author contributions

BL and WH completed data curation. FY and LW provided software support. NJ and ML provided experimental methodology. LL drafted the manuscript and drew the figures. BZ checked and modified the manuscript. All authors contributed to the article and approved the submitted version.
